# Using the Promise of Sonodynamic Therapy in the Clinical Setting against Disseminated Cancers

**DOI:** 10.1155/2015/316015

**Published:** 2015-08-25

**Authors:** Matthew Trendowski

**Affiliations:** Department of Biology, Syracuse University, 107 College Place, Syracuse, NY 13244, USA

## Abstract

Sonodynamic therapy (SDT) is a form of ultrasound therapy in which specialized chemotherapeutic agents known as sonosensitizers are administered to increase the efficacy of ultrasound-mediated preferential damage of neoplastic cells. Multiple *in vitro* and *in vivo* studies have indicated that SDT has the ability to exhibit profound physical and chemical changes on cellular structure. As supportive as the data have been, assessment of this method at the clinical level has been limited to only solid tumors. Although SDT has shown efficacy against multiple adherent neoplastic cell lines, it has also shown particular promise with leukemia-derived cell lines. Potential procedures to administer SDT to leukemia patients are heating the appendages as ultrasound is applied to these areas (Heat and Treat), using an ultrasound probe to scan the body for malignant growths (Target and Destroy), and extracorporeal blood sonication (EBS) through dialysis. Each method offers a unique set of benefits and concerns that will need to be evaluated in preclinical mammalian models of malignancy before clinical examination can be considered.

## 1. Introduction

**Figure 1:**
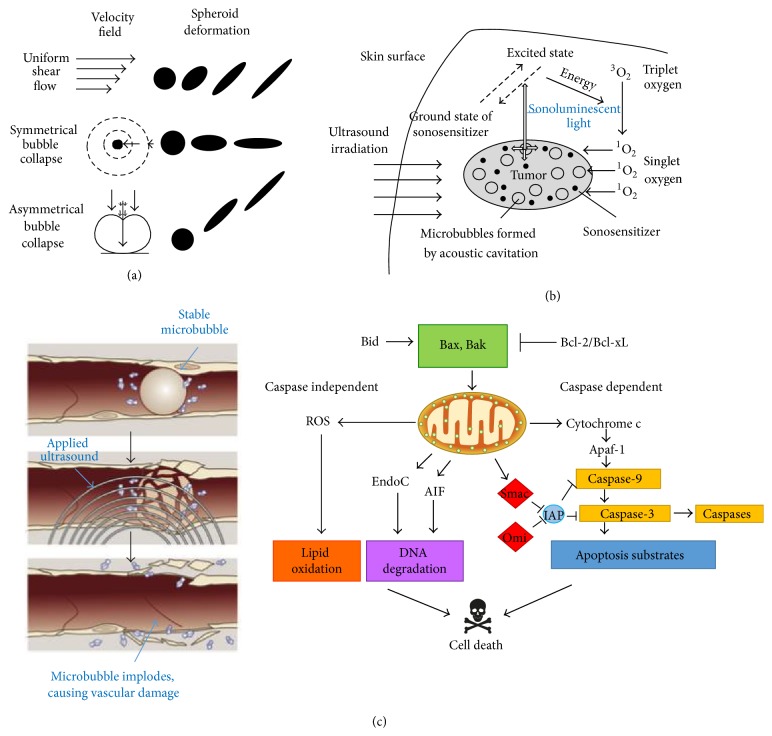
Antineoplastic mechanisms of ultrasound. (a) Microbubbles are unevenly stretched by ultrasonic waves, causing an unequal distribution of force known as inertial cavitation. Microbubbles oscillating in a stable motion reflect stable cavitation, while the expansion and contraction of microbubbles that are unequal and markedly exaggerated are indicative of inertial cavitation. Subsequent stress results in microbubble implosion, creating considerable amounts of energy. (b) The energy provided by the collapse of microbubbles potentiates the formation of sonoluminescent light within the cell. The light subsequently activates endogenous compounds within the cell that release ROS when returning to the ground state. (c) Many tumors rely on angiogenesis to sustain increased metabolic activity. Microbubbles can enter the tumor vasculature, and at sufficiently high amplitudes, ultrasound induces significant vascular damage, shutting down blood flow. The vessels develop and harbor hypoxic regions, causing oxidative stress; lack of nutrients and increased acidity induce apoptosis. In addition, malignant cells exposed to ultrasound often undergo apoptosis through the intrinsic pathway. Caspase-3 is upregulated by proteins such as Bax and Bak that integrate into the mitochondrial membrane, facilitating apoptotic signaling. It is important to note that sonosensitizers have been developed to significantly increase the efficacy of each mechanism. Images courtesy of [1].

Sonodynamic therapy (SDT) is a promising novel treatment modality that has yielded impressive anticancer effects in both* in vitro* and* in vivo* studies. It has been repeatedly demonstrated that ultrasound preferentially damages malignant cells based on the size differential between such cells and those of normal histology [1]. SDT is a form of ultrasound therapy in which specialized agents known as sonosensitizers are administered to increase the extent of preferential damage exerted by ultrasound against neoplastic cells (Figure 1). Preliminary studies examining the antineoplastic potential of sonosensitizers focused on the propensity of ultrasound to activate reactive oxygen species (ROS) producing agents, thereby eliciting oxidative stress that preferentially induced apoptosis in malignant cells [2–5]. Since then, the list of potential sonosensitizers has grown tremendously and has diversified to include cytoskeletal-directed agents, echo contrast agents, and vascular disrupting agents [1]. Multiple comprehensive literature reviews have been compiled that provide detailed explanations of the mechanisms that allow SDT to preferentially damage neoplastic tissue under conditions that do not notably perturb normal cells [1, 6–9]. These references comprehensively review the mechanisms illustrated in Figure 1 and should be referred to for further explanation.

Recent studies have indicated that ultrasound-mediated cancer therapies can potentiate notable antineoplastic activity against a variety of malignancies at the clinical level [10–13]. As such, using ultrasound to promote drug synergy between chemotherapeutic agents in an attempt to preferentially damage malignant cells appears feasible. In fact, SDT has notable similarities to photodynamic therapy (PDT), a proven method that is currently used in the clinical setting in the treatment of various skin carcinomas and other epithelial tumors [14]. The major difference between SDT and PDT is the energy source used to activate the chemotherapeutic agent (sound versus light). In PDT, light is used to excite porphyrins and other endogenous molecules that emit photoluminescent light after returning to the ground state [14]. Such activity produces excess levels of ROS that inflict substantial damage on the cellular integrity of malignant cells, eventually inducing apoptotic mechanisms. While PDT has indeed shown efficacy against particular squamous carcinomas, the effective range of the treatment does not extend far past the skin barrier [1], thereby limiting the utility of PDT in oncology.

By contrast, SDT uses ultrasound that can easily penetrate deep tissue layers where some malignancies reside. Although it retains the ability to produce ROS in the form of sonoluminescent light, SDT does so through inertial cavitation, the process of creating microbubbles in liquids, such as cellular cytoplasm. When microbubbles implode, they give off substantial amounts of energy (in upwards of 5000 K and 800 atm), a phenomenon that produces sonoluminescent light, subsequently leading to the production of ROS [6]. The energy released from microbubble implosion also severely damages malignant cells through hydrodynamic shear forces, destroying vital cytoskeletal structures of cells that already have a severely perturbed cytoskeleton due to dysplasia and subsequent anaplasia [1, 8]. Further, the synergistic effects of SDT and sonosensitizers other than ROS agents are not replicated in PDT, as light does not inflict damage through as many mechanisms as does sonication. While PDT can effectively activate ROS agents and other species dependent on a light activating source, cytoskeletal alterations and perturbed tumor vasculature networks are atypical. Therefore, PDT is also limited in the variety of sensitizing agents that are available. SDT inflicts damage on malignant cells through multiple mechanisms, providing the opportunity to utilize a vast array of antineoplastic agents [1]. This could significantly reduce the frequency of drug resistant tumors found within patients, as cells would have to overcome the various mechanisms elicited by SDT.

A prerequisite to clinical testing is evidence of* in vivo* efficacy. Ultrasound-mediated chemotherapy, such as improved drug delivery through sonoporation, SDT, or both, has indeed demonstrated notable efficacy against multiple forms of malignancy (Table 1). Since SDT is a novel treatment modality that has only recently begun clinical investigation, no standardized treatment approaches currently exist. Therefore, this paper intends to inform clinicians and academics about potential protocols that could be used to effectively administer SDT in the clinical setting, particularly in the treatment of leukemia. It should be noted that this is a preliminary attempt to devise effective treatment methods for a novel antineoplastic therapy and substantial revisions will likely be necessary. However, by bringing SDT to the forefront of clinical discussion, it may one day be possible to use this treatment modality as an effective method to treat a substantial variety of malignancies found in patients.

## 2. Current Methods of Ultrasound-Mediated Cancer Therapy

Although the primary focus of this paper will be on SDT, there is a diversity of methods in which ultrasound can be used to supplement cancer therapeutic protocols, many of which are currently being clinically examined. These methods will be briefly reviewed to demonstrate the utility of ultrasound-mediated approaches in the treatment of neoplasms. This will also enable a proper comparison of current ultrasound-mediated cancer therapies with SDT, thereby demonstrating why combining antineoplastic agents to supplement the mechanisms of ultrasonic damage is such a sensible prospect. It should be noted that SDT is not intended to supplant these currently used ultrasound-mediated cancer therapies. Rather, it should be used alongside such approaches to supplement the therapeutic use of ultrasound, as well as other antineoplastic interventions.

### 2.1. Improving Drug Delivery through Sonoporation

While microbubbles are often systemically injected into patients to improve diagnostic imaging, such structures develop naturally when ultrasound is applied [15, 16]. When microbubbles form under low intensity ultrasound, their oscillations are capable of increasing the permeability of microvessels, thereby enhancing cellular uptake of molecules, nanoparticles, and therapeutic agents. The increased permeability is typically due to sonoporation, the temporary opening of pores in the plasma membrane generated by microbubbles oscillating in a stable motion, known as stable cavitation [17, 18]. Sonoporation has been shown to be an effective method to improve drug uptake, and work to promote the delivery of anticancer agents into tumor tissue through microbubble potentiated microvascular permeability enhancement is being investigated in many* in vivo* experiments [1, 8, 9, 19]. Such research has been motivated by the fact that the effectiveness of many chemotherapeutic agents is limited by the inability to reach therapeutic concentrations within tumor tissue. Low intensity ultrasound is ideal for sonoporation, as it potentiates a steady increase of stabilized microbubbles within the cell, facilitating delivery of small molecules to the cytosol [11]. It has even been shown that ultrasound improves virotherapeutic approaches in oncology as it increases the efficacy of gene transfer to the directed tumor target [20–22].

### 2.2. Permeating the Blood Brain Barrier with Ultrasound to Increase Drug Delivery

Primary brain tumors, as well as metastatic growths from other cancers that have migrated to the organ, present a major challenge to chemotherapy. The blood brain barrier (BBB) is a highly selective permeability barrier that separates the circulating blood from the brain extracellular fluid (BECF) in the central nervous system (CNS) [23, 24]. It presents a formidable obstacle to the movement of many molecules as the normal brain capillaries have tight interendothelial junctions, few pinocytotic vesicles, and no fenestrations or small openings. As a consequence of this substantial impermeability, the principle route by which drugs and other molecules cross the BBB is by simple diffusion. However, many antineoplastic agents are large and/or hydrophilic molecules that are incapable of being carried across the BBB through this process [24]. Even if the drug is successful in crossing the BBB in likely diminished concentrations, many brain tumors are encapsulated by tumor microvessel populations that constitute a blood tumor barrier (BTB), providing an additional layer of protection [23–25]. Although highly lipophilic chemotherapeutic agents (lomustine/CCNU and temozolomide) are able to readily cross the BBB [26, 27], many malignancies of the brain (particularly glioblastoma multiforme) have particularly dim prognoses [28], even when drugs are used concomitantly with radiotherapy [29, 30]. While improvement to intrathecal administration directly into the arachnoid membrane of the brain or spinal cord has seen considerable progression in recent years (cytarabine, hydrocortisone, and methotrexate have been approved for such use [31–33]), numerous complications with the procedure still exist.

However, many of the issues with trying to successfully administer chemotherapeutic agents into the CNS may be reduced by temporarily increasing the permeability of the BBB through the use of high intensity focused ultrasound (HIFU). In particular, recent advances have enabled delivery of HIFU through intact human calvaria with magnetic resonance imaging (MRI) guidance. Such technology has been shown to improve essential tremor occurrences in patients treated with MRI-guided focused ultrasound thalamotomy (selected ablation of thalamic regions) [34, 35]. Multiple studies have shown that low intensity ultrasound can significantly increase the permeability of the BBB, without harming any cellular structures [36–40]. Such a discovery is pivotal for clinical applications of HIFU, as temporarily permeating the BBB allows previously unavailable chemotherapeutic agents or other drugs to be used in novel medical applications, particularly when combined with echo contrast agents that produce microbubbles to facilitate delivery [41, 42].

### 2.3. High Intensity Focused Ultrasound Ablates Solid Tumors

In addition to demonstrating potential as a method to temporarily permeate the BBB, HIFU is also currently being investigated to treat malignant growths due to its propensity to induce thermal ablation [9, 43]. Destruction of malignant growths through induced heating has achieved noticeable success through the use of percutaneous radiofrequency ablation (RFA), the direct placement of one or more radiofrequency electrodes into the tumor tissue [44, 45]. Temperatures between 60 and 100°C are then generated by a high frequency alternating current, which induces frictional heating when ions in the tissue attempt to follow the changing directions of the alternating current [46]. A similar effect can be achieved by microwave ablation (MWA), in which electromagnetic waves generate heat, thereby also destroying cells by direct hyperthermic exposure [47, 48].

However, HIFU offers a distinct advantage over other methods of thermal ablation in that it is the only noninvasive hyperthermic modality. To potentiate hyperthermic conditions, multiple ultrasound beams are focused on a selected focal area to generate temperatures of 60°C or higher through the use of acoustic energy, inducing coagulative necrosis in the targeted tissue [44]. HIFU also offers a novel mechanism by which to damage hyperthermic cells, as inertial cavitation causes uncontrolled expansion and contraction of gaseous nuclei in the cytoplasm, thereby leading to the collapse of the cell and nuclear membranes, as well as vital subcellular organelles (mitochondria and endoplasmic reticulum) [49, 50]. While HIFU has demonstrated clinical efficacy in a variety of cancer types [49, 51, 52], it is most widely employed in the treatment of prostate adenocarcinoma [53]. Although the actual efficacy of HIFU in prostate adenocarcinoma therapy has been questioned in recent years [53–55], it still remains as a viable treatment option through clinical trials.

### 2.4. Endoscopic Ultrasound-Guided Fine Needle Injection

Endoscopic ultrasonography (EUS) has been used in the clinical setting for more than 30 years and was originally developed to diagnose pancreatic diseases, as well as carrying out malignancy staging. It has since become vital for pancreatic adenocarcinoma diagnosis, as EUS is the most sensitive nonoperative imaging test for the detection of malignant pancreatic lesions, with a reported sensitivity between 87 and 100% [56]. However, development of linear array echoendoscopes has enabled the incorporation of fine needle injection (FNI) to potentiate a novel therapeutic approach. Recently, EUS-FNI has been clinically used for the localized delivery of anticancer agents (including small molecules and biotherapeutics) into targeted tumors [57–59]. This form of localized administration offers potential advantages over more traditional systemic chemotherapy, as it theoretically minimizes unwanted toxicity, while potentially increasing therapeutic concentrations at the tumor site. In addition, EUS-FNI can be used to locally administer ablating agents, such as ethanol, directly into pancreatic lesions to induce cell death by causing cell membrane lysis, protein denaturation, and vascular occlusion [60]. Interestingly, EUS can be used to guide physicochemical therapies as well, such as interstitial brachytherapy [61], PDT [62, 63], and RFA [64, 65], suggesting potentially novel avenues for cancer therapy.

## 3. Targeting Leukemia with Sonodynamic Therapy

SDT has shown notable efficacy against a variety of neoplastic cell lines* in vitro* and* in vivo* (Table 2) and is now under preliminary examination at the clinical level for the treatment of solid tumors [66–69]. However, the potential of this therapeutic intervention against disseminated cancers has not been as extensively explored, despite the fact that many hematological malignancies show considerable sensitivity to the sonochemotherapeutic approach, particularly leukemias of both myeloid and lymphoid origin. Therefore, the potential application of SDT against leukemia will be the primary focus of this paper.

Leukemia is unique to cancer biology as it is inherently metastatic [70]. Leukocytes are required to move throughout the vascular system, indicating that no mutation is required for anchorage independent growth. This helps explain why some leukemias (particularly acute lymphoid leukemia: ALL) are common pediatric malignancies as less fundamental alterations are required for neoplastic transformation [70]. However, leukemia is also unique in that it typically does not form primary tumor sites but rather saturates the vasculature with aberrant cells, eventually compromising the immune system, blood clotting, and erythrocyte transport [70]. As such, leukemia cells are often freely floating alongside healthy blood cells. Being in such close proximity to cells that are vital for normal physiological functioning, it seems appropriate that SDT should have the capability to preferentially damage the malignant cells, while leaving healthy cells intact. While normal human hematopoietic stem cells (hHSCs) can sometimes be rescued through hematopoietic stem cell transplantation (as has been shown following high dose chemotherapy [71, 72]), studies have confirmed that SDT can preferentially damage leukemia cells in the presence of normal blood cells. As will be discussed later, the size differential between leukemic and normal blood cells can be dramatically increased using appropriate antineoplastic agents.

## 4. Applying Sonodynamic Therapy in the Clinical Setting

Seeing that SDT has yet to be clinically evaluated against disseminated cancers, there has been no analysis as to how this treatment modality could be practically applied for these malignancies. Although SDT fundamentally relies on an ultrasound system, there are a variety of ways in which the generated ultrasound can be delivered. The three procedures that the author believes to be the most salient for leukemia therapy are heating of the appendages as ultrasound is applied to these areas (Heat and Treat), using an ultrasonic probe to scan the body for malignant growths (Target and Destroy), and extracorporeal blood sonication (EBS) through dialysis. Each method offers a unique set of benefits and concerns that will need to be evaluated in preclinical mammalian models of malignancy prior to being given clinical consideration. Finding proper frequency ranges and sound intensities for ultrasound will also be of clinical importance, but considerable work in this area has been done at the clinical level in the treatment of solid tumors [66–69], as well as in preclinical mammalian models (Table 1), and such data should be readily extrapolated for the treatment of leukemia.

### 4.1. Heat and Treat

The vascular system is truly a remarkable piece of biological architecture, as it provides a reliable solution to the oxygenation of tissues far away from the thoracic cavity where the heart and lungs reside. Blood cells are unique among the great diversity of cell types used in physiological functioning as they are required to move throughout the entire body in a timely manner. In fact, the approximate 5.6 L of blood within the body circulates the entire cardiovascular system in one minute [73]. As such, most blood will pass through the extremities in a short amount of time. Therefore, sonicating the arms and legs of patients could be a potential method for sonicating circulating leukemia cells.

Heat comes into play as it has been shown to increase the efficacy of specific chemotherapeutic treatments that could be used in SDT. Mild hyperthermia (39–43°C) is an adjuvant therapy that has yielded substantial benefits in the treatment of a variety of tumor types. Hyperthermia increases tumor blood flow and vascular permeability [74, 75], thereby promoting drug delivery to the targeted site [76–78], which is essential for effective SDT treatments. The slight increase in temperature enhances the uptake and efficacy of numerous antineoplastic agents, particularly platinum based compounds, resulting in increased cytotoxicity [79–81]. In addition to these biological responses, hyperthermia has been shown to be an effective drug-release trigger for temperature-sensitive nanoparticles, resulting in an improved and more targeted drug delivery system [82, 83]. The degree of thermal enhancement of hyperthermia is inherently dependent on the ability to localize and maintain therapeutic temperature elevations. Due to the often heterogeneous and dynamic properties of tissues (most notably blood perfusion and the presence of thermally significant blood vessels), therapeutic temperature elevations are difficult to spatially and temporally control [84]. Ultrasound can provide an additional role as the heat source for the treatment, as it has been shown to permeate a higher degree of spatial and dynamic control of heating compared to other commonly used heating modalities [84]. These advantages include a favorable range of energy penetration characteristics in soft tissue, as well as the ability to shape the energy deposition patterns [84].

The setup for Heat and Treat would be relatively straightforward. The patient could be placed in a chair, while ultrasonic devices are attached to the forearms and/or near the ankles of the patient. To reduce the potential of erythrolysis and thromboses, it would likely be advantageous to move the ultrasonic devices to different locations along the appendages after a given amount of time. Multiple ultrasonic systems could be used to produce hyperthermia in addition to activating the sonosensitizers, or alternatively other methods of inducing hyperthermia could be applied such as immersing the patient's hands and feet in hot water. Nevertheless, some form of ultrasound is inherent in this procedure as it is the sound energy (sonication) that inflicts preferential damage on malignant cells through diverse mechanisms of action. The sonosensitizers used in the treatment would be administered intravenously (i.v.) before sonication, allowing the chemotherapeutic agents to accumulate in the bloodstream. Once an effective dosage has been applied, the patient would be connected to the ultrasonic devices, necessitating a waiting period between application of sonosensitizers and ultrasound. The applied ultrasound could be run continuously or in short bursts during the treatment. The length of each individual SDT treatment remains unclear and would have to be determined by clinicians after initial trials. However, the simplicity and relatively low potential risks to the patient during Heat and Treat provide compelling reasons for using sonication in the clinic. Although incidental normal blood cell (erythrocytes, leukocytes, megakaryocytes, and thrombocytes) destruction may be a potentially hazardous issue, it can be monitored by attending clinicians to ensure that preferential damage is indeed occurring.

### 4.2. Target and Destroy

Although Heat and Treat is a potential avenue for treating leukemias and other hematological malignancies, sonicating the appendages will yield little benefit for other cancers that are often concentrated at a primary tumor site. Further, some patients have leukemia cells that remain trapped in the bone morrow and would therefore be inaccessible to the Heat and Treat method. This is commonly seen with aleukemic cells that remain in the bone marrow, severely perturbing normal production of blood cells. Without the luxury of using the vascular system to transport blasts to areas that could be more readily sonicated, another sonication approach needs to be devised, one that is capable of scanning the body for concentrated pockets of malignant cells and then sonicating such sites with high intensity ultrasound.

In fact, such technology already exists and could be applied in the clinic with a few minor adjustments. Ultrasonic probes are devices capable of delivering high frequency or high intensity ultrasound to localized areas and are commonly used in the medical world for diagnostic imaging (high frequency) or even breaking up calculi (high intensity). Medical ultrasonography uses a substantial variety of ultrasonic probes, and many operational systems are available for testing with SDT. In fact, such probes are currently being used in the clinic for extracorporeal shock wave lithotripsy (ESWL). Breaking up formed calculi in the gall bladder or kidney with ultrasound requires considerable intensity. The lithotripter used in such procedures breaks up stones with tolerable collateral damage by using an externally applied, focused, high intensity acoustic pulse [81, 82], which could readily be converted for SDT-mediated treatment protocols. ESWL can actually be seen as a proof of concept of SDT, as it breaks up calcified deposits through inertial cavitation, just as malignant cells are in SDT.

Due to the advances in medical imaging, it is now possible to readily locate primary tumor sites, providing the basis for Target and Destroy SDT procedures. By injecting sonosensitizers i.v. or subcutaneously (s.c.) at tumor aggregates prior to treatment, ultrasonic probes can be locally applied to the affected site, thereby allowing a potentially site-specified chemotherapeutic approach. Although it may not apply directly to leukemia, combining the drug activation of SDT with the specificity of EUS-FNI may be particularly beneficial for solid tumors, such as pancreatic adenocarcinomas. As such, this therapeutic method has apparent clinical implications outside hematological malignancies, as a great diversity of cancers could be treated using Target and Destroy. However, the true diversity of cancers that can be treated through this form of SDT will only be determined through preclinical and eventual clinical evaluation.

### 4.3. Extracorporeal Blood Sonication

While Heat and Treat and Search and Destroy have potential clinical utility, both treatment methods inherently rely on ultrasonic waves traveling through the skin barrier, as well as complex internal structures. As such, ultrasound loses some of its intensity as it travels through the human body. Instead of increasing the wattage to obtain the same amount of intensity, if there was a way to remove malignant cells from the body so they could be treated in an extracorporeal environment, there would be no sound inhibitors protecting such cells from sonication.

**Figure 2:**
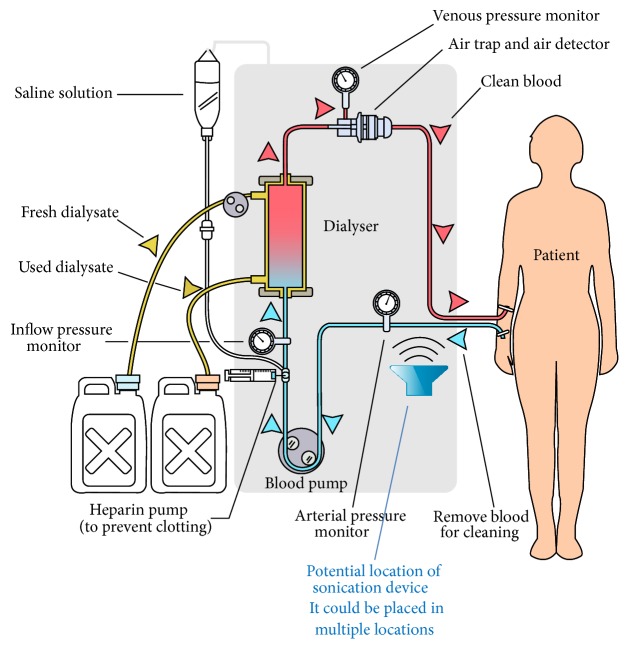
Extracorporeal blood sonication. Hemodialysis requires the patient's blood to be pumped outside of the body into an extracorporeal setting. This provides an opportunity for leukemia cells to be sonicated without sound attenuation from anatomical structures, as ultrasound can be applied to the dialysis tubing. Sound intensities would likely be reduced as there is only a tube standing in the way between the ultrasonic waves and the patient's blood.

Although such an approach is unfeasible for most malignancies, leukemia is unique in that it does not form a primary tumor site. However, its most beneficial asset can potentially be exploited as a profound fatal flaw. Since most leukemias are localized in the blood, it would be rather straightforward to draw the malignant cells out of the body through dialysis. While dialysis is typically used on patients to act as an artificial replacement for lost kidney function due to renal failure, it could also be employed to treat leukemia in an extracorporeal setting. Sonosensitizers can be injected i.v. as in the previous two procedures, with roughly the same amount of time passing before injection and sonication. The patient would then undergo a typical hemodialysis procedure in which blood is pumped outside of the body, thereby removing the natural sound barriers of human anatomy (Figure 2). There would be nothing standing in the way between the malignant cells and the ultrasonic waves that are able to elicit marked preferential damage. In effect, this SDT procedure allows an* in vivo* setting to become almost* in vitro*. Since the* in vitro* studies of SDT with leukemia have yielded notable results [85–90], this may be an effective method by which to administer ultrasound to patients.

Further, EBS provides additional benefits in that preoptimization of the treatment before administration and prevalidation of its efficacy are feasible prospects. A small sample of the patient's blood could be drawn and exposed to the proposed sonochemotherapeutic protocol. Subsequent cytometric analysis of the treated sample could then ascertain the success and selectivity of the approach. After validation of the proposed protocol, the patient could then be plugged into the dialysis system for systemic treatment. Such an initial check will enable clinicians to fine-tune or dramatically alter the antineoplastic agents and/or ultrasound settings being administered, thereby increasing patient safety and treatment efficacy.

However, it may be the case that the sound intensities used for Heat and Treat and Search and Destroy will be inappropriate for EBS. There is very little standing in the way between the blood and the high intensity ultrasound being administered. While normal blood cells are more resistant to SDT, they are not invulnerable. Sufficient sound intensities will cause just as much damage to these cells as the malignant cells that are within close proximity [23]. Therefore, the sound intensity used in EBS might have to be considerably reduced. Nevertheless, it still provides the most direct route for sonicating leukemic cells within the patient. This method could potentially be used in combination with Target and Destroy, so that malignant cells caught within the bone marrow are preferentially damaged as well. EBS might also be used to sonicate metastatic cells in the blood that inadvertently become dislodged from a primary tumor due to physical agitation from focused ultrasound if SDT is ever used to treat solid malignancies. It may even be feasible to use all three treatments (Heat and Treat, Target and Destroy, and EBS) in a comprehensive scanning and removal of leukemia cells found within the patient.

## 5. Sonosensitizer Treatment

Multiple studies have indicated that sonosensitizers often damage malignant cells through a variety of mechanisms. While sonosensitizers are grouped by their differences in primary mechanism of action, many of these drugs have additional mechanisms that are similar or even the same as other types [1, 8]. Therefore, it is possible that a combination of sonosensitizers would have a substantial synergistic effect when combined with ultrasound, as this energy form has been shown to damage cells by similar mechanisms. Such concomitant therapy is necessary for SDT to stand out as a viable clinical approach, since it will come across a substantial variety of neoplastic growths, each capable of different methods of overcoming applied treatments.

There are currently several promising sonosensitizers available for further* in vivo* characterization. Although only specific potential combinations will be mentioned here, the full spectrum of these chemotherapeutic agents can be found in a previous review, which provides a comprehensive analysis of sonosensitizers, as well as their mechanisms of preferential damage [1]. Being able to develop treatment regimens in which the synergistic effects of different sonosensitizers are applied can be potentially vital for clinical applications. Such treatments could substantially amplify the capability of ultrasound to preferentially damage malignant cells, therefore decreasing the rate at which drug resistance is observed.

By default, malignant cells have a perturbed cytoskeleton due to the effects of dysplasia and subsequent anaplasia. With so many alterations present in malignant cells, the cytoskeleton provides an ideal opportunity to attain preferential damage. Specifically, one of the most intriguing possibilities is to preferentially damage malignant cells based on their considerable size differential in comparison to their normal counterparts. This phenomenon gives rise to the concept of substantially enlarging neoplastic cells to increase their already noticeable size differential with normal cells.

Cytochalasin B is a mycotoxin that disrupts the actin cytoskeleton and inhibits cytokinesis by interfering with formation of the contractile ring as well as the development of the cleavage furrow [1]. While malignant cells are unable to divide, they continue to form nuclei and eventually become enlarged and multinucleated. Such cells have more DNA targets, increasing the likelihood of apoptosis when combined with a DNA-directed agent, as demonstrated by my laboratory using DOX against P388/ADR murine leukemia cells [91], as well as O'Neill with cytarabine against BHK/IV^3^ hamster renal tumor cells [92]. Further, the multinucleated cells have a large cell volume, making them more susceptible to direct cell destruction by physical agitation. Preferential damage of malignant cells is facilitated by the fact that normal cells exposed to cytochalasin B exit the cell cycle and enter the G_0_ state until sufficient actin levels are restored [93, 94]. Therefore, only malignant cells that have lost the ability to enter this resting phase become enlarged and multinucleated, providing ideal targets for sonication.

To put the size differentials into perspective, normal erythrocytes are typically between 6 and 8 *μ*m and leukocytes range between 10 and 15 *μ*m (the occasional macrophage grows up to 20 *μ*m). By contrast, cytochalasin B-treated U937 human monocytic leukemia cells have been shown to grow in excess of 20 *μ*m, with some reaching nearly 40 *μ*m in diameter [89, 95]. Such cells have reduced cytoskeletal integrity and are attractive targets for sonication. Further, it has been shown that cytochalasin B substantially increases mitochondrial activity, opening up the opportunity to use ROS agents that specifically target the organelle. U937 cells even show a marked reduction in clonogenicity after being exposed to cytochalasin B treatments [89, 90], thereby inhibiting the most prolific phenotypic characteristic of cancer, aberrant cell proliferation.

While cytochalasin B-alone treatments could yield substantial results on leukemia patients when combined with ultrasound, the fact that affected cells become profoundly multinucleated provides the opportunity for concomitant chemotherapy with a nucleic acid-directed agent. One of particular note is doxorubicin as it has been shown to produce a much higher concentration of ROS when applied in combination with ultrasound, enabling the chemotherapeutic agent to damage doxorubicin-resistant cell lines [1]. Such effects were derived from a cell line shown to be resistant to doxorubicin-alone control treatments, further substantiating the amplifying effect sonosensitizers have in SDT. There has been a similar effect when ultrasound/doxorubicin treatments were applied to U937 cells, suggesting the agent can be effective against multiple leukemia cell lines when used concomitantly with ultrasound [1, 89]. This phenomenon of potentially reversing doxorubicin resistance has also been shown in athymic mice inoculated with HepG2 multidrug resistant hepatocellular carcinoma cells, as mice had an average 62% reduction in tumor volume a month later [96]. Such results provide* in vivo* evidence of the ability of SDT to increase the efficacy of antineoplastic agents.

In addition, cytochalasin B-mediated ultrasonic sensitivity may be further potentiated through the use of microtubule-disrupting agents, such as the vinca alkaloids. My laboratory has shown that the mechanisms by which neoplastic cells are enlarged can influence ultrasonic sensitivity [90]. Microfilament- and microtubule-disrupting agents substantially increase the ultrasonic sensitivity of U937 cells (cytochalasin B and vincristine, resp.). By contrast, agents that stabilize microfilaments or microtubules (jasplakinolide and paclitaxel, resp.) do not potentiate the same effect. Further, we have demonstrated that concomitant cytochalasin B/vincristine treatments profoundly decrease U937 cell clonogenicity after ultrasound exposure [90]. The synergy between cytochalasin B and vincristine has been previously reported by Kolber and Hill [97], suggesting that concomitant use of microfilament- and microtubule-disrupting agents in concurrence with ultrasound may elicit substantial drug synergy.

Combining ultrasound with cytochalasin B and microtubule-directed as well as nucleic acid-directed agents may be notably efficacious when applied in the clinical setting due to the mechanisms by which ultrasound damages malignant cells. However, the results of such treatments obtained in* in vitro* and* in vivo* studies might be considerably diminished when actually applied in the clinic. If preliminary clinical studies determine that ultrasound combined with cytochalasin B-mediated concomitant chemotherapy is not effective, there are a tremendous variety of other sonosensitizers that are currently available for therapeutic evaluation. However, if initial trails are successful, further refinements could be made to determine conditions optimal for inhibiting leukemia cell proliferation. As with any novel treatment, the only way to determine actual efficacy is to give the therapeutic approach real world experience.

## 6. Conclusion

SDT appears to be a viable approach to preferentially damaging malignant cells in the clinical setting. Ultrasound by itself can produce antitumor effects under appropriate conditions, as exhibited by HIFU. However, such effects are not always widespread and tumor populations can be refractory or develop resistance to ultrasound alone treatments. That is why SDT is such a sensible prospect, as it significantly enhances the efficacy of sonication, while still displaying preferential damage towards malignant cells. Every mechanism by which ultrasound destroys malignant tissue can in fact be amplified when an appropriate sonosensitizer is administered. Such drugs often damage cells through multiple mechanisms, creating a potential synergistic effect when sonosensitizers of different classes are used in combination. Nevertheless, SDT has yet to be clinically evaluated against leukemia, and appropriate methods by which to administer ultrasound have not been formalized.

Due to the similarities between metastatic cancer cells and leukemia [70], it is likely that SDT could be applied in the clinical setting to preferentially damage circulating metastases through an ultrasound-mediated treatment protocol. While HIFU is potentially useful against solid tumors, it works by concentrating multiple ultrasonic beams on a particular site of diseased tissue. Therefore, this approach is less feasible for disseminated cancers, as is the case with leukemia. As opposed to HIFU, which focuses ultrasound at a given neoplastic growth, SDT could be used to successfully target disseminated cancers using the proposed EBS treatment model. Successful treatment could have a marked influence on progression-free survival, as complications from metastases result in more than 90% of cancer mortality [98, 99]. As shown by ESWL for calculi removal, ultrasound has the propensity to fragment large chemical aggregates. Since carcinomas often circulate as metastatic emboli to avoid the unsuitable environment of the circulatory system [100], it seems likely that ultrasound could be used for breaking up such aggregates, thereby exposing the cells to the unsuitable environment. Without the protective embolism, it is likely that most metastatic cells in circulation would die, significantly reducing the likelihood of disease migration. While this would not account for micrometastases that have already reached the intended secondary site, patients can always be monitored after treatments have concluded to protect against such occurrences.

The idea of combining ultrasound with drugs that amplify the ways in which it preferentially damages malignant cells is gaining more legitimacy as successful studies have validated the potential of SDT. With preliminary clinical evaluations currently underway, data necessary to ascertain its actual antineoplastic activity will be acquired in the near future, thereby determining whether SDT warrants further study as a novel form of cancer therapy.

## Figures and Tables

**Table 1:** Efficacy of sonodynamic therapy *in vivo*.

*In vivo* parameters	*In vivo* efficacy	Class of sonosensitizer	Primary mechanism of sonosensitizer
Doxorubicin-loaded microbubbles (DOX-MBs) were administered intravenously in Lewis rats while one of the two tumors (pancreatic carcinomas) was exposed to ultrasound (1.3 MHz; mechanical index 1.6). DOX tissue concentration was measured in tumors and control organs after the experiment [101].	All rats survived the DOX-MB administration without any sign of embolisation/occlusion of the pulmonary vasculature. Ultrasound targeted destruction of DOX-MBs resulted in a 12-fold higher tissue concentration of DOX and a significantly lower tumor growth in the target tumor compared to the contralateral control tumor.	DOX: anthracycline, ROS agent	It intercalates DNA, preventing DNA replication and protein synthesis, has been shown to reverse drug resistance in drug resistant K562/A02 leukemia cells [85], and produce ROS after ultrasound activation [102]

**Metronomic cyclophosphamide (MCTX) was employed and administered through drinking water to athymic mice that harbored MDA-MB-231 breast cancer tumors. Ultrasound stimulated microbubble treatments were conducted at 1 MHz employing short bursts (0.00024 duty cycles) at 1.6 MPa in combination with the commercial microbubble agent Definity** [103].	**The USMB induced an acute reduction of blood flow as confirmed with US contrast imaging and DiOC7 perfusion staining. Longitudinal experiments demonstrated that significant growth inhibition occurred in MCTX-only and USMB-only treatment groups relative to control tumors. The combined USMB and MCTX treatment group showed significant growth inhibition and survival prolongation relative to the USMB-only and MCTX-only treatment groups.**	**MCTX: vascular disrupting agent, alkylating agent, Definity: echo contrast agent (increases microbubble concentration)**	**MCTX alkylates guanine nucleotides, inhibiting DNA replication and protein synthesis**; **the agent is converted in the liver to an active form for chemotherapeutic effects; and increased microbubbles from Definity**, **thereby amplifying inertial cavitation**

A novel porphyrin-derived sonosensitizer designated DEG (7,12-bis(1-(2-(2-hydroxyethoxy)ethoxy)ethyl)-3,8,13,17-tetramethylporphyrin-2,18 dipropionatomanganese) was injected into SCID mice xenograft models with MKN-74 gastric cancer cells, followed by ultrasound (1.0 MHz, 1.0 W/cm^2^ output intensity, and 10% duty cycle for 1-2 min) [104].	SDT with DEG three times a week for 2 weeks potently inhibited tumor growth compared to ultrasound only or no treatment. It was shown that ROS are generated and mediate sonotoxicity of ultrasound with DEG on MKN-74 cells.	DEG: ROS agent, porphyrin	It generates ROS after excitation from sonoluminescent light that disrupts mitochondrial membrane potential, loss of electrochemical gradient, causes cristae to fragment, and induces apoptotic cascade to trigger caspase proteases

Epirubicin hydrochloride (EPI) inhibition on tumor growth by ultrasound was tested using five-week-old male nude mice injected s.c. with HL-60 human promyelocytic leukemia cells. 1-MHz ultrasound and 3 W/cm^2^ output power density were applied through aquasonic coupling gel for 30 s to the tumor region of a mouse [86].	Ultrasound applied locally to the tumor resulted in a substantially increased drug uptake in tumor cells. The inhibition on tumor growth depended on the position of drug injection and phospholipid-based microbubble (PMB) application. Artificial sonoporation nuclei significantly enhanced transient pore formation on cell membranes which facilitates outside drugs entry into the cells.	EPI: ROS agent, anthracycline	It generates ROS after excitation from sonoluminescent light that disrupts mitochondrial membrane potential, loss of electrochemical gradient, causes cristae to fragment, and induces apoptotic cascade to trigger caspase proteases

**The taxane docetaxel (Taxotere) was used for evaluating SDT as it has previously been shown to have potent antitumor effects when combined with small molecule vascular disrupting agents. Experiments were conducted on PC3 human prostate cancer cell tumors implanted in athymic mice. USMB treatments were performed at a frequency of 1 MHz employing sequences of 50 ms bursts (0.00024 duty cycles) at 1.65 MPa. USMB treatments were administered on a weekly basis for 4 weeks with docetaxel (DTX) being given intravenously at a dose level of 5 mg/kg** [105].	**The USMB treatments, either alone or in combination with DTX, induced an acute reduction in tumor perfusion, accompanied by significantly enhanced necrosis and apoptosis after 24 hours. Longitudinal experiments showed a modest prolongation in survival but no significant growth inhibition occurred in DTX-only and USMB-only treatment groups relative to control tumors. The combined USMB-DTX treatment group produced tumor shrinkage in weeks 4–6 and significant growth inhibition and survival prolongation relative to the control, USMB-only, and DTX-only treatment groups. **	**DTX: cytoskeleton agent, taxane**	**It stabilizes GDP-bound tubulin polymers, thereby inhibiting mitosis**

The sonodynamically induced antitumor effect of porfimer sodium (PF) was evaluated on a chemically induced mammary tumor in Sprague-Dawley rats. The timing of 24 hours after the administration of PF was chosen for the ultrasonic exposure, based on pharmacokinetic analysis of the PF concentrations in the tumor, plasma, skin, and muscle. The rats were exposed to ultrasound (3 W/cm^2^) for 15 min [106].	The synergistic effect between PF administration and ultrasonic exposure on the tumor growth inhibition was significant. The ultrasonic intensity showed a relatively sharp threshold for the synergistic antitumor effect, which is typical of an ultrasonic effect mediated by acoustic cavitation. Therefore, a marked synergistic effect between PF administration and ultrasonic exposure on the tumor growth inhibition was observed at a PF dose of 2.5 mg/kg and at a free-field ultrasonic intensity of 3 W/cm^2^.	PF: ROS agent, hematoporphyrin derivative	It generates ROS after excitation from sonoluminescent light that disrupts mitochondrial membrane potential, loss of electrochemical gradient, causes cristae to fragment, and induces apoptotic cascade to trigger caspase proteases

5-Aminolevulinic acid (ALA), a precursor to the ROS agent protoporphyrin IX (PpIX) was investigated for its antiangiogenic potency *in vivo*. SAS human oral cancer cell suspensions were injected s.c. into the flanks of BALB/c mice. ALA was intraperitoneally injected into mice in the ALA and ultrasound + ALA groups at a dose of 250 mg/kg body weight. After 4 hours of administration of ALA, the mice were placed on a plexiglass plate with the tumor immersed in degassed water. Tumors were irradiated by ultrasound (1.1 MHz, 2 W/cm^2^, 50% duty cycle) for 5 min [107].	Ultrasound treatment significantly decreased microvessel density (MVD) compared with control, and the reduction of MVD was more prominent in the ultrasound + ALA group. Accordingly, the expression level of VEGF, a critical proangiogenic factor, was reduced in tumors treated with ultrasound irradiation. Ultrasound plus ALA induced more significant decrease in VEGF expression than ultrasound alone. It also inhibited the secretion of VEGF in SAS cells more significantly in the presence of ALA.	ALA: ROS agent, precursor to hematoporphyrin derivative	It generates ROS after excitation from sonoluminescent light that disrupts mitochondrial membrane potential, loss of electrochemical gradient, causes cristae to fragment, and induces apoptotic cascade to trigger caspase proteases

Reversal of DOX resistance was investigated in a study of low intensity ultrasound. Athymic nude mice were inoculated with HepG2 multidrug resistant hepatocellular carcinoma cells. Ultrasound with pulsed irradiation (0.5 W/cm^2^) was administered for 10 min to both ultrasound/DOX and ultrasound only groups [96].	Ultrasonic treatment resulted in an average 62% reduction in tumor volume a month later. The relative levels of MDR1 and MRP were dramatically reduced in ultrasound/DOX groups, suggesting a reversal of drug resistance.	DOX: anthracycline, ROS agent	It intercalates DNA, preventing DNA replication and protein synthesis, ROS agent

The study was conducted on CT26 colon carcinoma tumors in BALB/c mice. In the respective groups, protoporphyrin IX (PpIX) or the gold nanoparticle-protoporphyrin IX conjugate was injected into the tumors. Ultrasound irradiation (1.1 MHz, 2 W/cm^2^, 3 min) was performed on the tumors 24 hours after injection [108].	A significant difference in the average relative volumes of the tumors 13 days after treatment was found between the ultrasound + gold nanoparticle-protoporphyrin IX group and the other groups. The longest doubling and 5 folding times were observed in the ultrasound + gold nanoparticle-protoporphyrin IX and ultrasound + protoporphyrin IX groups.	PpIX: ROS agent hematoporphyrin derivative	It generates ROS after excitation from sonoluminescent light that disrupts mitochondrial membrane potential, loss of electrochemical gradient, causes cristae to fragment, and induces apoptotic cascade to trigger caspase proteases

**C57BL/6J female mice were inoculated s.c. with Hepa1-6 hepatocellular carcinoma cells. Herpes simplex virus thymidine kinase under the control of kinase domain-containing receptor (KDR, angiogenic growth factor's corresponding receptor) promoter was used for targeted gene therapy. Plasmid DNA with or without microbubble contrast agent of SonoVue was intravenously injected. Ultrasound (1 MHz, 2 W/cm** ^**2**^ **, 5 min) was delivered to hepatic carcinomas in mice. The KDR-tk gene transfer was followed by ganciclovir injection for 10 days and then the diameters of tumors were measured every 4 days for 28 days** [109].	**Compared with the group treated by ultrasound alone, KDR-tk gene** **treatment** **by ultrasound combined with SonoVue restrained tumor growth and increased survival time of tumor-bearing mice; microvessel density in group mediated by ultrasound and SonoVue was significantly lower than that in ultrasound alone group. An apoptosis index increased in the group treated by ultrasound and SonoVue compared with the group treated by ultrasound alone, whereas there was no significant difference between group mediated by SonoVue alone and phosphate-buffered saline alone group.**	**SonoVue: echo contrast agent **	**It increases microbubbles in systemic circulation to enhance effects of inertial cavitation, substantially increasing the efficacy of viral gene transfer**

Bold print refers to studies that demonstrated efficacy only through improved drug delivery and not ultrasound-mediated activation of chemotherapeutic agents.

**Table 2:** Sonosensitizers tested in sonodynamic therapy.

Sonosensitizer	Class	Primary mechanism	Synergistic effect with ultrasound
Doxorubicin (Adriamycin)	Anthracycline	It intercalates DNA, preventing DNA replication and protein synthesis	Increased efficacy with multidrug resistant K562/A02 cells at 20 kHz, 0.25 W/cm^2^, 60 s intervals [85]; similar effects observed with U937 cells [62]; the agent can be used in tandem with HMME on U937 cells for a greater effect [88]

Cytochalasin B	Cytoskeleton agent	It disrupts actin cytoskeleton and prevents cytokinesis by interfering with formation of the contractile ring as well as the cleavage furrow; cells do not divide and become grossly enlarged and multinucleated	Sonic sensitivity was increased in U937 cells when cytochalasin B was administered at 1.5 *μ*M; cells often grew to 20 *μ*m or greater and were unable to tolerate ultrasound treatments in which normal blood remained stable; increased mitochondrial activity and reduced clonogenicity were also observed [89]; cytochalasin B sensitized multiple leukemia cell lines (U937, THP1, K562, Molt-4, and L1210) to 20 kHz and 23.5 kHz ultrasound [90]

Methotrexate	Cytoskeleton agent, antimetabolite agent	It causes competitive inhibition of dihydrofolate reductase, an enzyme that participates in tetrahydrofolate synthesis, prevents production of thymidine as well as all purine bases, and causes the same effects on the cytoskeleton as cycloplatin	Aberrant features with the cytoskeleton of HeLa cells were observed using 1.8 MHz, 0.22 W/cm^2^ [110]

Cisplatin	DNA alkylating agent	It alkylates guanine nucleotides, preventing DNA synthesis	Increased cytotoxicity to multiple cancer types; ultrasound replenishes labile cells lost due to treatments [6]

Diaziquone	DNA alkylating agent	It alkylates guanine nucleotides, preventing DNA synthesis	Increased cytotoxicity to multiple cancer types; ultrasound has the same effects on depleted labile cells [6]

Albunex	Echo contrast agent	It increases microbubbles in systemic circulation to enhance effects of inertial cavitation	Macrocytic (grossly enlarged) erythrocytes were damaged by the increased proportion of microbubbles at intensities that left normal erythrocytes intact using 1.15 MHz, 3 MPa, indicating SDT preferentially damages based on size [15, 111]

Levovist	Echo contrast agent	It increases microbubbles in systemic circulation to enhance effects of inertial cavitation; cells exposed to drug treatment have low mitochondrial membrane potential, high superoxide production, increased intracellular calcium concentration, and phosphorylation of histone H2AX after sonication	Multiple leukaemia cell lines (Jurkat, Molt-4, U937) were significantly damaged Using 1 MHz, 0.3 W/cm^2^, 10% duty factor pulsed at 100 Hz [112]

ATX-S10	ROS agent, porphyrin	It has a substantially longer sonoluminescent lifetime than other porphyrin agents, providing more opportunity to generate singlet oxygen; it follows the same mechanism as other porphyrins	Inhibited growth of colon-26 cells injected into athymic mice [6]

Hematoporphyrin monomethyl ether (HMME)	ROS agent, porphyrin	It generates singlet oxygen that disrupts mitochondrial membrane potential, loss of electrochemical gradient, causes cristae to fragment, and induces apoptotic cascade to trigger caspase proteases	Significant destruction of U937 cells with 1 MHz, 1 W/cm^2^, 60 s intervals, increases intracellular singlet oxygen content [113], showing synergistic effect with DOX as both produce ROS [88]

All agents were shown to have increased efficacy due to ultrasound-activating mechanisms.
